# Regional Alteration within the Cerebellum and the Reorganization of the Cerebrocerebellar System following Poststroke Aphasia

**DOI:** 10.1155/2022/3481423

**Published:** 2022-03-22

**Authors:** Xiaotong Zhang, Zhaocong Chen, Na Li, Jingfeng Liang, Yan Zou, Huixiang Wu, Zhuang Kang, Zulin Dou, Weihong Qiu

**Affiliations:** ^1^Department of Rehabilitation Medicine, The Third Affiliated Hospital of Sun Yat-sen University, Guangzhou, Guangdong Province, China; ^2^Department of Radiology, The Third Affiliated Hospital of Sun Yat-sen University, Guangzhou, Guangdong Province, China

## Abstract

Recently, an increasing number of studies have highlighted the role of the cerebellum in language processing. However, the role of neural reorganization within the cerebellum as well as within the cerebrocerebellar system caused by poststroke aphasia remains unknown. To solve this problem, in the present study, we investigated regional alterations of the cerebellum as well as the functional reorganization of the cerebrocerebellar circuit by combining structural and resting-state functional magnetic resonance imaging (fMRI) techniques. Twenty patients diagnosed with aphasia following left-hemispheric stroke and 20 age-matched healthy controls (HCs) were recruited in this study. The Western Aphasia Battery (WAB) test was used to assess the participants' language ability. Gray matter volume, spontaneous brain activity, functional connectivity, and effective connectivity were examined in each participant. We discovered that gray matter volumes in right cerebellar lobule VI and right Crus I were significantly lower in the patient group, and the brain activity within these regions was significantly correlated with WAB scores. We also discovered decreased functional connectivity within the crossed cerebrocerebellar circuit, which was significantly correlated with WAB scores. Moreover, altered information flow between the cerebellum and the contralateral cerebrum was found. Together, our findings provide evidence for regional alterations within the cerebellum and the reorganization of the cerebrocerebellar system following poststroke aphasia and highlight the important role of the cerebellum in language processing within aphasic individuals after stroke.

## 1. Introduction

Aphasia is one of the common complications among individuals after left-hemispheric stroke, and it has been reported that approximately 40% of survivors from left-hemispheric stroke are diagnosed with aphasia [1]. Patients with poststroke aphasia (PSA) have multiple aspects of language impairments, including naming, comprehension, and spontaneous speaking. Because patients with PSA mainly have difficulty communicating with others, it is difficult for them to function in society, thus decreasing the quality of life of patients and financially burdening society [2, 3]. For such reasons, investigating the disease-specific mechanism following PSA is important because it helps improve the present understanding of the language process after stroke and refine the therapeutic strategy for aphasia treatment. Although reorganization of the language network within the cerebral cortex after PSA has been widely studied in recent years [4, 5], few studies currently focus on the reorganization of the cerebrocerebellar system within aphasic individuals, while accumulating evidence hints at the potential role of the cerebellum in language processing.

The concept of a “linguistic cerebellum” is currently drawing attention. On the one hand, patients with cerebellar impairments have shown different aspects of cognitive impairments, including language processing. It has been discovered that patients with resected right cerebellar tumors and individuals who survive right cerebellar infractions tend to show linguistic problems [6]. Among adolescents diagnosed with autism spectrum disorder, decreased activations within the cerebellum during language tasks were found. Also, these patients exhibited reduced functional connectivity between right Crus I and distributed supratentorial language areas [7, 8]. Jeong et al. also found that microstructural impairment of the cerebrocerebellar circuit may be relative to communication problems [9]. On the other hand, studies based on task-based functional magnetic resonance imaging (fMRI) revealed that the cerebellum participated in different aspects of language processing [10–13], and the activation areas during language tasks were mainly located in right Crus I/Crus II, lobule VI, and midline lobule VIIAt, which are characteristic areas of right lateralization [14]. Diffusion imaging analysis also found structural connections linking the supratentorial language areas and the cerebellum [15, 16], and the structural frontal-cerebellar loop might contribute to verbal working memory [17]. Such findings indicated that the cerebellum plays a role in language processing via informative interaction through the cerebrocerebellar circuits, and it is believed that considering the cerebellum when constructing the language network will make the network more accurate [18]. However, whether regional alterations within the cerebellum and functional reorganization of cerebrocerebellar circuits occur within aphasic individuals after stroke remains unknown.

Motivated by the evidence and the limitations mentioned above, in this study, we used structural and resting-state functional magnetic resonance imaging (fMRI) to investigate the structural and functional alterations within the cerebellum and to explore the functional reorganization of the cerebrocerebellar system among patients with aphasia after left-hemispheric stroke. First, voxel-based morphometry (VBM) analysis was used to explore the structural alterations of gray matter volume (GMV) within the cerebellum, accompanied by the calculation of regional spontaneous brain activity by using the amplitude of low-frequency fluctuation (ALFF) as an indicator. Second, regions with significant differences in the VBM analysis were used as regions of interest (ROIs) in the following functional connectivity (FC) analysis with the goal of exploring aberrant FC in the cerebrocerebellar system. Additionally, the Granger causality analysis (GCA) was used to study alterations in effective connectivity (EC) within the cerebrocerebellar circuit. We considered that GCA could be used to recognize changes in the directionality of information flow [19] and thus provide comprehensive evidence for functional reorganization of the cerebrocerebellar system following PSA. We hypothesized that PSA would not only cause changes in regional structure and neural activity in cerebellar language-related areas but also result in functional reorganization within the cerebrocerebellar language system.

## 2. Methods

### 2.1. Subjects

Patients who were diagnosed with aphasia after left-hemispheric stroke at the rehabilitation department of the 3^rd^ Affiliated Hospital of Sun Yat-sen University Guangzhou city, China, as well as age- and sex-matched healthy controls (HCs), were recruited for this study. The diagnosis of aphasia was based on the language test criteria from the Chinese version of the Western Aphasia Battery (WAB); patients recruited in this study were native Chinese speakers and had normal language function before stroke. To eliminate heterogeneity, patients with bilateral hemispheric stroke or who had a history of other cerebrovascular or mental diseases were excluded from this study. To ensure the safety of MRI scanning, participants with MRI contraindications were also excluded.

### 2.2. Language Assessment

Before undergoing MRI scans, each patient's language severity was assessed by the same professional language therapist using the WAB. WAB scores include aphasia quotient (AQ), representing the overall severity of language impairment, and four subtypes, including naming, repetition, comprehension, and spontaneous speech.

### 2.3. MRI Acquisition

All the participants recruited in the present study underwent MRI scanning with a GE 3.0-Tesla scanner (General Electric Company, USA) in the radiology department of the 3^rd^ Affiliated Hospital of Sun Yat-sen University. rs-fMRI data were first acquired with the gradient-echo EPI sequence: repetition time (TR) = 2000 ms, echo time (TE) = 35 ms, flip angle = 90°, thickness = 4 mm, slice number = 35, field of view = 240 × 240 mm^2^, 3.75 mm × 3.75 mm in-plane resolution, and 243 time points for a total of 486 s. During scanning, participants were informed to fixate on the black cross presented on the screen against a white background and avoid thinking of anything. Structural MRI data were acquired with a high-resolution, axial magnetization-prepared rapid gradient echo (MPRAGE) T1-weighted sequence: TR = 8200 ms, TE = 3.2 ms, flip angle = 90°, field of view (FOV) = 256 × 256 mm, slice thickness = 1.2 mm, and voxel size = 1 × 1 × 1 mm^3^. Foam paddings were placed inside the head coil to reduce head motion, and earplugs were also used for noise reduction.

### 2.4. Data Preprocessing

For VBM processing, structural images were processed with the use of the CAT12 toolbox (CAT12 Version 12.6; http://dbm.neuro.uni-jena.de/cat/), which was implemented in SPM12 (SPM12; http://www.fil.ion.ucl.ac.uk/spm/software/spm12/) running in the MATLAB 2020a environment. All T1 images were first manually quality checked by the researchers followed by bias-field inhomogeneities correction. Then, the individual T1 images were segmented into three tissue components, including gray matter (GM), white matter (WM), and cerebrospinal fluid (CSF), using tissue probability maps (TPMs). Total intracranial volume (TIV) was also calculated by adding total GM, WM, and CSF volumes. Afterward, native-space tissue segments were registered to the standard Montreal Neurological Institute (MNI) template by using the DARTEL algorithm. The normalized GM images were subsequently modulated to preserve the original tissue volumes in each voxel and then smoothed with a 6 mm Gaussian kernel with a full width at half maximum (FWHM).

rs-fMRI images were processed with the use of the RESTplus toolbox (RESTplus v1.24; http://restfmri.net/forum/restplus) and SPM12 within the MATLAB 2020a environment. The first 10 time points were excluded to eliminate the influence of participants' adaptation to the scanning noise, followed by slice timing and realignment for the correction of head motion. Before the following spatial normalization, any participant who had maximum translation larger than 3.0 mm or maximum rotation larger than 3.0 degrees was excluded from the rs-fMRI analysis. Spatial normalization was then performed so that the individual images would be standardized into MNI space. After these procedures, functional images were then smoothed with a 6 mm FWHM Gaussian kernel. The linear trend of the time series was removed, and the nuisance signal (Friston's 24 head motion) was regressed out.

### 2.5. GMV Analysis

To explore the alteration of GMV within the cerebellum, an independent two-sample *t* test was used for voxelwise group comparisons with the use of the spatially unbiased infratentorial template (SUIT, http://www.diedrichsenlab.org/imaging/suit_download.htm), which is a spatially unbiased template of the human cerebellum [20], and age and TIV were used as covariates. The Gaussian random-field (GRF) correction (voxel level: *p* < 0.001, cluster level: *p* < 0.05) was used for multiple comparison correction, and we considered *p* < 0.05 statistically significant. The MNI coordinates of the peak *t*-value from regions with significant differences between groups were then extracted as the center of ROI for the following analysis. The radius of each ROI was 6 mm.

### 2.6. rs-fMRI Analysis

#### 2.6.1. Cerebellar ALFF Analysis

Regional ALFF was analyzed to study the regional spontaneous neural activity within the regions with significant GMV differences in participants in the patient group. The whole-brain ALFF map of each individual was first calculated. To improve the normality of the data, each individual mean ALFF (mALFF) map was calculated by dividing the ALFF of each voxel by the global average value. The regional mALFF value of each participant was then extracted within each spherical ROI.

#### 2.6.2. Cerebrocerebellar FC Analysis

A seed-based FC analysis was used to analyze FC alterations in the cerebrocerebellar circuit. The average time series of each ROI was extracted, followed by the calculation of the Pearson correlation coefficients between the time series of the ROI and every other voxel within the whole brain. The FC map of each subject was then standardized to a zFC map with Fisher's *r*-to-*z* transformation to improve normality.

#### 2.6.3. GCA

GCA has been thought to be a useful method to investigate EC among discrete regions without prior knowledge [21]. In the theory of GCA, if the past time series of *X* and *Y* predict the current time series of *X* more accurately than the past *X* time series itself, then *Y* has a “causal influence” on *X*, and vice versa [22]. In the present study, we defined the time series of the ROI as the time series of *X* and the time series of each other voxel within the whole brain as the time series of *Y*. The definition of ROIs was the same as the FC analysis. Voxelwise, coefficient-based GCA was performed with the use of the RESTplus toolbox, and the EC map of each individual was created, followed by Fisher's *z* transformation (zGC) to improve normality.

### 2.7. Statistical Analysis

We used the RESTplus package, mentioned previously, for the statistical analysis of GMV, zFC, and zGC data. For the zFC and zGC data, an independent two-sample *t* test was used for voxelwise group comparisons within the whole brain. We used the GRF correction (voxel level: *p* < 0.001, cluster level: *p* < 0.05) for multiple comparison correction, and *p* < 0.05 was considered statistically significant.

Demographic and regional mALFF data analysis was performed with the IBM SPSS statistics 26. The Shapiro-Wilk test was used to evaluate the normality. For continuous variables, if the data were normally distributed, an independent two-sample test was used to evaluate the difference between groups; otherwise, the Mann–Whitney *U* test was used. For the classified variables, Fisher's precision probability test was used to assess between-group differences. Spearman's correlation analysis was also used to explore the correlation between WAB scores and values of mALFF values, between WAB scores and zFC values, and between WAB scores and zGC values. We considered *p* < 0.05 to indicate statistical significance.

## 3. Results

### 3.1. Demographic Data and Language Performance

**Figure 1:**
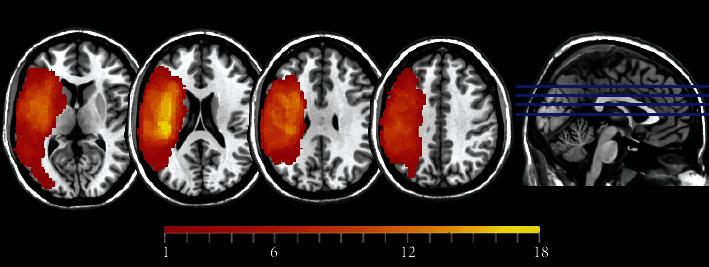
Lesion overlap of 20 PSA patients. The color bar represents the number of patients with a lesion in a specific voxel (maximum 18 out of 20).

We finally recruited 20 PSA patients and 20 HCs in the present study. There was no significant difference between age, sex, or education years, and detailed information is shown in Table 1. The lesion overlap of the recruited patients is shown in Figure 1.

### 3.2. Cerebellar GMV Alteration

**Figure 2:**
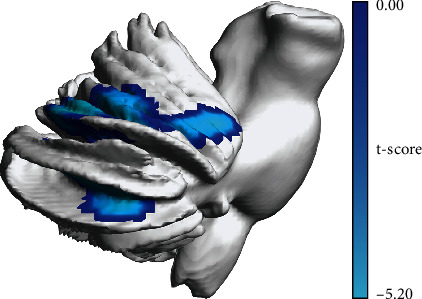
Resulting clusters of VBM analysis.

**Figure 3:**
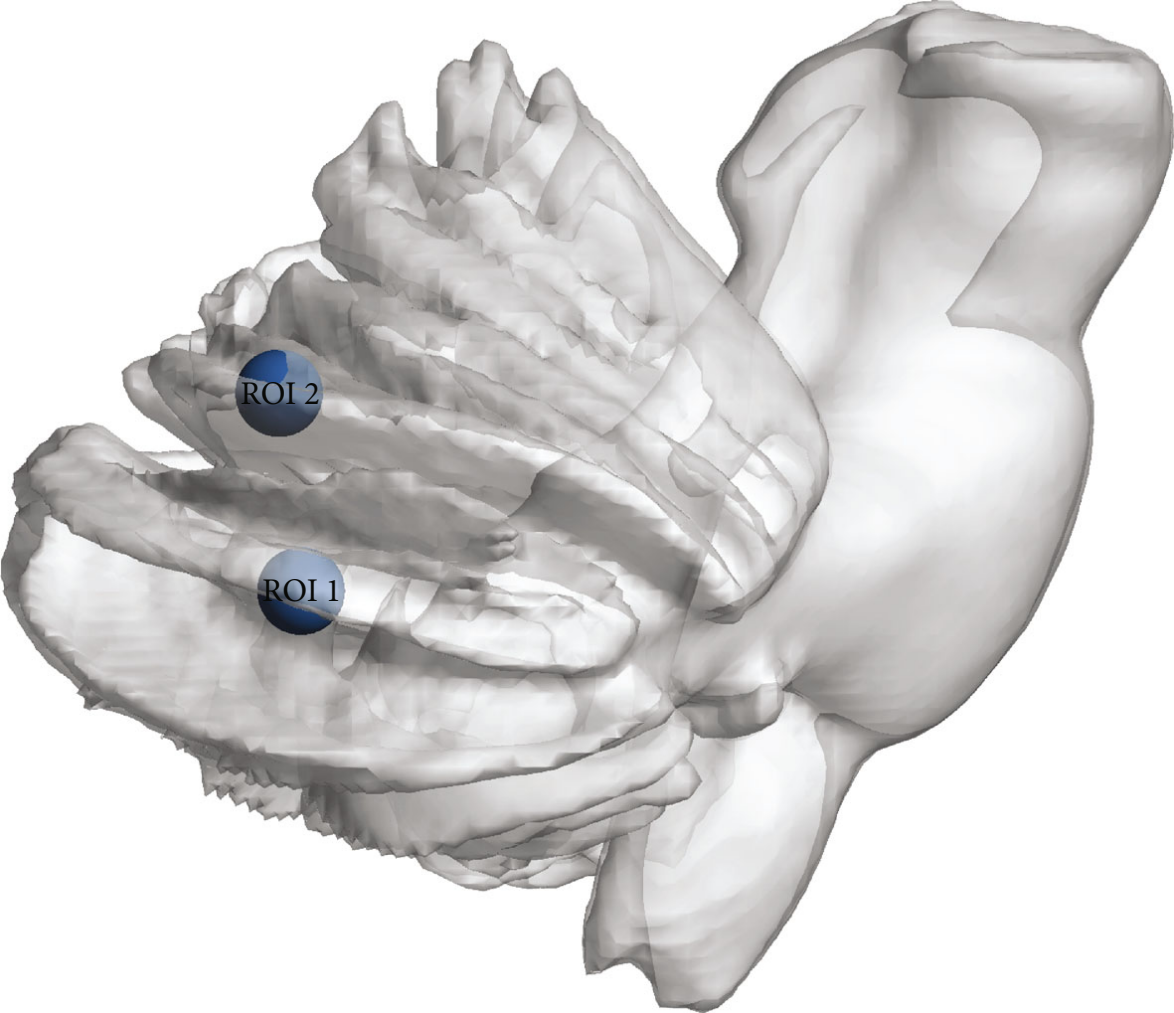
The MNI coordinates of the peak *t*-value from regions with significant between-group differences in VBM analysis were extracted as the center of ROI. ROI 1 (36, -67.5, and -36): rCrus I; ROI 2 (22.5, -69, and -22.5): rLobule VI.

The VBM analysis showed that the GMV of two clusters in the right cerebellum, centering at right cerebellum Crus I (rCrus I) and lobule VI (rLobule VI), of patients was significantly lower than that of HCs. (Table 2, Figure 2). The MNI coordinates of the peak *t*-value from regions with significant differences between groups were then extracted as the center of ROI for the following analysis. The radius of each ROI was 6 mm (Figure 3).

### 3.3. The Results of rs-fMRI Analysis

Two patients were excluded from the rs-fMRI study for head motion, leaving 18 patients in the following re-fMRI analysis.

#### 3.3.1. ALFF Result

**Figure 4:**
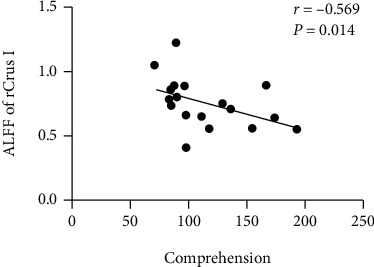
Correlation analysis between ALFF values of rCrus I and comprehension scores (*r* = −0.569, *p* = 0.014).

There was no significant difference in ALFF values within ROIs between participants in the two groups. Correlative analysis revealed that the ALFF value in rCrus I was negatively correlated with comprehension scores in individuals in the patient group (Table 3, Figure 4).

#### 3.3.2. Results of Seed-Based FC Analysis

**Figure 5:**
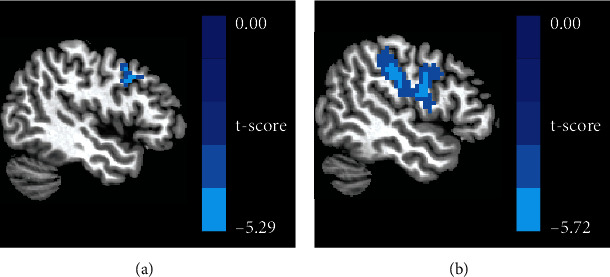
With the use of ROI 1 and ROI 2 as the seed in seed-based FC analysis separately, the result showed decreased FC (a) between the rCrus I and the lPreCG, as well as (b) between the rLobule VI and the lPreCG.

**Figure 6:**
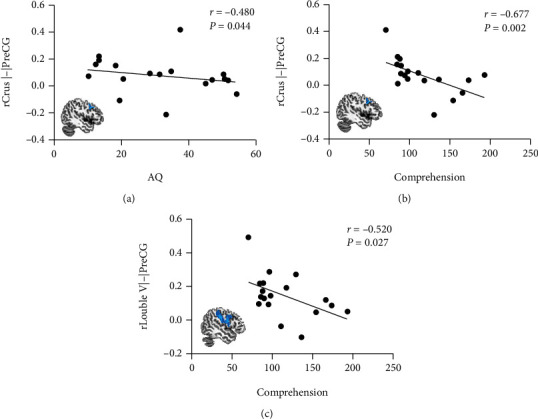
(a) Negative correlation between rCrus I-lPreCG and AQ scores (*r* = −0.480, *p* = 0.044). (b) Negative correlation between rCrus I-lPreCG and comprehension scores (*r* = −0.677, *p* = 0.002). (c) Negative correlation between rLobule VI-lPreCG and comprehension scores (*r* = −0.520, *p* = 0.027).

Aphasic individuals showed significantly lower FC between rCrus I and the left precentral gyrus (lPreCG) as well as between rLobule VI and the lPreCG than participants in the HC group (Table 4, Figure 5). Correlation analysis revealed that reduced FC between rCrus I and the lPreCG was significantly correlated with AQ (*r* = −0.480, *p* = 0.044) and comprehension (*r* = −0.677, *p* = 0.002) scores. In addition, a negative correlation between comprehension scores and FC between rLobule VI and the lPreCG was observed (*r* = −0.520, *p* = 0.027) (see Figure 6).

#### 3.3.3. The Results of GCA

**Figure 7:**
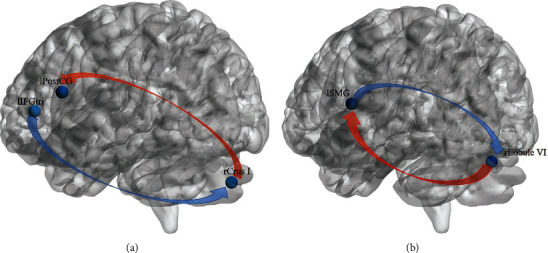
Aberrant effective connectivity from and to rCrus I as well as from and to rLobule VI. (a) Using rCrus I as an ROI, decreased EC from the lIFGtri to rCrus I and increased EC from rCrus I to the lPostCG were found. (b) Using rLobule VI as an ROI, decreased EC from the lSMG to rLobule and increased EC from rLobule VI to the lSMG were found.

The results of voxelwise GCA are shown in Table 5 and Figure 7. Compared with participants in the HC group, patients with PSA demonstrated decreased EC from rCrus I to the left postcentral gyrus (lPostCG) and from rLobule VI to the left supramarginal gyrus (lSMG). Additionally, the left inferior frontal gyrus pars triangularis (lIFGtri) illustrated a higher causal influence on the rCrus I; also, the lSMG demonstrated an increased causal influence on rLobuleVI among aphasic individuals.

## 4. Discussion

In the present study, we investigated structural and functional alterations within the cerebellum, as well as functional reorganization of the cerebrocerebellar system following aphasia caused by left-hemispheric stroke. Our results demonstrated that among aphasia patients after stroke, GMVs in rCrus I and rLobule VI were significantly lower than those in HCs, while there was no significant difference in ALFF values of those two regions between individuals in the two groups. FC analysis revealed reduced FC between rCrus I and the lPreCG as well as between rLobule VI and the lPreCG. In addition, there was enhanced EC from rCrus I to the lPostCG as well as from the lSMG to rLobule VI, whereas EC from the lIFGtri to rCrus I and from rLobuleVI to the lSMG was decreased. Moreover, FC and ALFF values are negatively correlated with WAB scores. To our knowledge, this is the first study to explore structural and functional reorganization of the cerebellum in addition to the reorganization of functional cerebrocerebellar circuits following PSA.

### 4.1. Structural and Functional Alterations within the Cerebellum

Changes in regional GMVs were demonstrated subsequent to aphasia after stroke. This phenomenon may be the result of the extension of anatomical lesions from the supratentorial area, as previous studies indicated that regions distant from the anatomical lesion tend to show structural alterations if these regions were in the same network [23, 24]. An increasing number of studies have confirmed that rLobule VI and rCrus I are activated during variations in language performance, suggesting that these two regions are part of the language network and play an important role in normal language processing. Accompanied by correlation findings from the present study, demonstrating that the value of ALFF in rCrus I were negatively correlated with language performance, we verified the participation of the cerebellum in language processing in aphasic individuals. Another explanation of the structural damage in the cerebellum may be caused by crossed cerebellar diaschisis (CCD). CCD is the phenomenon of decreased blood flow and metabolism within the contralateral cerebellum after supratentorial structural impairment and can be commonly recognized [25–27]. It has been reported that patients diagnosed with CCD often show cerebellar hypofunction with an intact cortical structure [24, 28], but some patients in the chronic stage may further experience cerebellar atrophy [29, 30]. However, although cerebellar atrophy was demonstrated in participants in the patient group, we did not find a decrease in ALFF values, which is one of the indicators of regional spontaneous neural activity, in these atrophic regions compared with HCs. Considering that the calculation of the ALFF value of each ROI is the average value within the region, the significant difference may be offset by the decreased GMV in the resulting regions. Moreover, the patients recruited in our study were mainly subacute aphasic patients whose GMV of the cerebellar cortex might be more likely to decrease after long-term hypometabolism; thus, we assumed that regional ALFF reductions in rCrus I and rLobule VI might be recognized if the aphasic individuals were in the acute stage after stroke.

### 4.2. Changes of Cerebrocerebellar FC

Reduced FC between rCrus I and the lPreCG, as well as between rLobule VI and the lPreCG, was observed in our study. The precentral gyrus, which is part of the dorsal stream in the language network, plays a role in mapping sound to meaning in the language process [31] and is thought to be related to phonological processing. It has been reported that patients with impairment of the precentral gyrus show inability in phonological judgment [32]; in addition, activation of the precentral gyrus during phonological tasks is well recognized among healthy individuals [33, 34]. Moreover, elderly individuals with hearing loss showed activation of several areas, including the precentral gyrus, when encountering complex sentences [35], suggesting that functional activity of the precentral gyrus is important in language processing when individuals already have impaired language ability. Combining accumulating evidence of the precentral gyrus in phonology and the notion that the frontal-cerebellar circuit is crucial in language processing, we assumed that the cerebrocerebellar circuits of rCrus I-lPreCG and rLobule VI-lPreCG are parts of the phonological network and influence language performance in aphasic individuals. Phonological processing is the procedure that understands spoken words by using language sounds [34, 36]. Our correlation analysis results showed that the FC between rCrus I and the lPreCG was negatively correlated with comprehension scores in the patient group, further verifying our assumption.

### 4.3. GCA

In the present study, aberrant EC between the cerebellum and language-related areas was also found, indicating the alteration of information flow within the cerebrocerebellar system following stroke. The inferior frontal gyrus (IFG) is considered the hub of the language network as it has a role in both phonologic and semantic processes. Studies based on lesion-symptom mapping (LSM) revealed that damage to the supramarginal gyrus and the postcentral gyrus among poststroke individuals is related to phonological deficits in single-word production [37–39]. A task-based fMRI study revealed that both regions participate in picture naming tasks among healthy individuals [40]. In general, these two areas together are thought to contribute to fluent speech production by phonetic-articulatory planning [41]. The postcentral gyrus, as a part of the sensorimotor system, also contributes to semantic processing, particularly action-related words [42]. In addition, an impaired structural connection between the postcentral gyrus and language-related areas due to the influence of a stroke on language performance [43] indicated the potential role of the postcentral gyrus in language processing.

Previous studies discovered that the severity of speech apraxia among patients was related to the degree of impairment of the supramarginal gyrus and the postcentral gyrus. Interestingly, in some cases, patients with only cerebellar damage showed similar symptoms of apraxia, suggesting that the disruption of the cerebrocerebellar circuit may cause the same outcome. Associative diffusion MRI analyses revealed that there are contralateral interconnections between the cerebellum and the cerebral cortex via the thalamus [16], and the dentate nucleus is structurally connected to various areas in the contralateral frontal lobe [15]. Such evidence may be the basis of information interplay between the cerebrum and the cerebellum in language processing. In the theory of cerebellar internal models, the cerebellum plays a role in motor speech planning by transmitting predictions on coming linguistic information and modifying speech execution if needed [44]. Through the dense structural connectivity between the cerebrum and the cerebellum, the linguistic cerebellum receives information from the frontotemporal area during speech production [45] and conveys predictive information to the prefrontal cortex to ensure accurate actual expression [46]. DCM is an alternative way to analyze the EC between regions; with the use of DCM, Sobczak found that the EC from lIFG to the pons and subsequently to the right superior cerebellum (rLobule VI/rCrus I) might contribute to verbal working tasks among healthy individuals [17]. They assumed that the right superior cerebellum might serve a role in the predictive mechanism [17]. Here, in the GCA results, we discovered that patients with aphasia had alterations in information flow both in the input and output stages within the internal model. We assumed that the decreased EC from the supratentorial area to the cerebellum might reflect the specific disease mechanism following PSA. When language-related areas, such as the IFG and supramarginal gyrus (SMG), are damaged, the information flow to the contralateral cerebellum might be subsequently reduced and might affect the information input to the cerebellar internal model and thus affect the expression of patients with aphasia. Furthermore, the increased EC from the cerebellum to the supratentorial areas might indicate compensation for the decreased informative interplay caused by a stroke and help individuals with language recovery. Consistent with our hypothesis, a study of the cerebellum as a neuromodulation target demonstrated that anodal tDCS to rCrus I helps language performance and increases FC between the cerebellum and the language hub [18, 47]. However, we noticed that cathodal tDCS to the right cerebellum also helped language recovery in some studies. The exact effect of tDCS intervention on the right cerebellum on the recovery of language in individuals with aphasia after stroke and the underlying mechanism of this effect are still worth exploring.

### 4.4. Limitation

There were limitations in the present study. First, this was an observational study with a relatively small sample, and it might be helpful to fully understand the specific mechanism within the cerebrocerebellar system after PSA with an enlarged sample size. Second, although the results of the study presented here showed functional reorganization within the cerebrocerebellar system after PSA, we did not explore the microstructural integrity of WM fibers between these areas. Notably, diffusion MRI is a useful technique that could be used to solve this problem and could be employed in future studies.

## 5. Conclusion

To our knowledge, this is the first article to explore neural reorganization within the cerebellum subsequent to PSA both from regional and integrative viewpoints. We discovered that PSA not only causes reduced matter volumes in right cerebellar lobule VI and Crus I but also induces aberrant functional connectivity as well as effective connectivity between the cerebellum and the supratentorial language-related areas. Together, this study may provide a new perspective to understand the reorganization of the cerebellum following aphasia caused by supratentorial lesion.

## Figures and Tables

**Table 1:** Demographic data and language performance.

	Patient group (*n* = 20)	Control group (*n* = 20)	*p*
Age	49.60 ± 9.30	52.05 ± 5.98	0.328
Sex (male/female)	18/2	16/4	0.661
Education (years)	12.10 ± 1.62	12.30 ± 1.84	0.717
Time from stroke (days)	65.25 ± 37.17	NA	NA
AQ	31.20 (31.95)	NA	NA
Naming	11.00 (20.75)	NA	NA
Repetition	37.00 (51.75)	NA	NA
Comprehension	98.00 (49.50)	NA	NA
Spontaneous speech	4.00 (6.50)	NA	NA

Data are presented as the mean ± standard deviation if the variables were normally distributed or presented as the median value (interquartile range) if the variables were the non-normally distributed. NA: not available. AQ: aphasia quotient.

**Table 2:** Between-group differences in VBM analysis (patient>control).

	Peak MNI coordinates				
Area	*x*	*y*	*z*	Voxel size	Peak *t*-value
rCrus I	36	-67.5	-36	813	-4.890
rLobule VI	22.5	-69	-22.5	1213	-5.195

**Table 3:** Between-group differences in ALFF within ROI 1 (rCrus I) and ROI 2 (rLobule VI).

	Patient group (*n* = 18)	Control group (*n* = 20)	*p*
ROI 1: rCrus I	0.760 ± 0.195	0.773 ± 0.127	0.805
ROI 2: rLobule VI	1.150 ± 0.385	1.226 ± 0.295	0.493

Data are presented as the mean ± standard deviation.

**Table 4:** Regions with significant between-group differences in seed-based FC analysis (patient>control).

			Peak MNI			
Seed	Region	Voxel size	*x*	*y*	*z*	Peak *t*-value
ROI 1: rCrus I	lPreCG	75	-51	9	33	-5.295
ROI 2: rLobule VI	lPreCG	317	-54	0	27	-5.723

**Table 5:** Regions with significant between-group differences in GCA (patient>control).

				Peak MNI			
		Region	Voxel size	*x*	*y*	*z*	Peak *t*-value
ROI 1: rCrus I	X2Y	lPostCG	1579	-63	-6	21	6.193
	Y2X	lIFGtri	646	-42	30	9	-7.387
ROI 2: rLobuleVI	X2Y	lSMG	204	-60	-24	15	5.692
	Y2X	lSMG	81	-54	-24	24	-5.185

X2Y: the causal outflow from the ROI (*X*) to the rest of the whole brain (*Y*); Y2X: the causal inflow to the ROI (*X*) from the rest of the whole brain (*Y*).

## Data Availability

The datasets used and analyzed in the present study are available from the corresponding author on reasonable request.
